# Post-warming embryo morphology is associated with live birth: a cohort study of single vitrified-warmed blastocyst transfer cycles

**DOI:** 10.1007/s10815-021-02390-z

**Published:** 2022-01-18

**Authors:** Meagan Allen, Lyndon Hale, Daniel Lantsberg, Violet Kieu, John Stevens, Catharyn Stern, David K. Gardner, Yossi Mizrachi

**Affiliations:** 1 Melbourne IVF, Melbourne, VIC Australia; 2 Reproductive Services Unit, The Royal Women’s Hospital, Melbourne, VIC Australia

**Keywords:** IVF, Vitrification, Grading, Warming, Blastocyst

## Abstract

**Purpose:**

This study aims to examine whether blastocyst morphology post-warming correlates with live birth.

**Methods:**

In this cohort study, morphological characteristics post-warming were reviewed in all single vitrified-warmed blastocyst transfer cycles performed between November 2016 and May 2017. Immediately before transfer, the degree of blastocoel re-expansion was graded as A, fully expanded; B, partially expanded ≥ 50%; C, partially expanded < 50%; and D, collapsed. The degree of post-warming cell survival was graded on a scale of 50 to 100% and was then classified into 4 groups: very low 50–70%, low 71–80%, moderate 81–90%, and high 91–100%.

**Results:**

Overall, 612 cycles were reviewed, of which 196 included PGT-A tested embryos. The live birth rate (LBR) increased from 11.4% in the collapsed blastocysts group to 38.9% in the post-warming full re-expansion group (*p* < 0.001) and from 6.5% for blastocysts with a very low cell survival rate to 34.7% for blastocysts with high cell survival rate (*p* = 0.001). LBR was 6.7% for blastocysts with the worst post-warming morphological characteristics, namely, collapsed with very low cell survival rate. On multivariate analyses, partial blastocyst re-expansion ≥ 50%, full re-expansion, and high cell survival rate remained significantly associated with live birth, after controlling for female age, pre-vitrification morphological grading, and PGT-A. A sub-analysis of cycles using PGT-A tested embryos showed similar results.

**Conclusion:**

Post-warming re-expansion and high cell survival rate are associated with higher LBR in euploid and untested blastocysts. However, embryos with poor post-warming morphology still demonstrate a considerable probability of live birth, and they should not be discarded.

**Supplementary Information:**

The online version contains supplementary material available at 10.1007/s10815-021-02390-z.

## Introduction

The utilization of frozen embryos in assisted reproductive technology (ART) has increased substantially over the last decade [1, 2]. The major forces behind this trend are improved embryo survival with vitrification method, the policy of single-embryo transfer employed in some clinics, the use of preimplantation genetic testing, and the use of freeze-all strategy for various indications [3].

Vitrification and warming pose great challenges to the embryo. Dehydration and rehydration might cause cellular damage. Moreover, the large volume of fluid contained in the blastocoel cavity exposes it to a potential risk of structural damage due to the formation of ice crystals, though to a lesser extent when using vitrification as compared with slow freezing. Most vitrified embryos will survive the vitrification-warming process. However, it has been shown that between 1 and 5% of vitrified blastocysts may fail to survive after warming [4, 5].

Pre-vitrification morphological grading is a well-established tool to estimate embryo integrity and predict treatment outcome [6]. The degree of blastocoel expansion and inner cell mass and trophectoderm differentiation were found to correlate with cycle outcome in fresh cycles [7], as well as in vitrified-warned embryo transfer cycles [8, 9]. However, less evidence exists regarding the predictive value of post-warming blastocyst characteristics. Some researchers have found that the degree of post-warming blastocyst re-expansion and cell survival correlated with cycle outcome [8, 10–13], while others have not [9, 14]. The European Society of Human Reproduction and Embryology (ESHRE) report on ART laboratory performance indicators selected the degree of re-expansion as the best post-thaw parameter for the prediction of live birth [15]. However, this was only based on a single study [8].

Previous studies that examined post-warming characteristics have had several limitations; some included cycles in which two or more embryos were transferred [10, 12, 16]. Hence, it was impossible to correlate specific characteristics with cycle outcome. Hong et al. included cycles with cleavage stage embryo transfer [16], in which post-warming re-expansion could not be assessed. Most studies did not report the live birth rate [10, 11, 14, 16]. Finally, to the best of our knowledge, the predictive value of post-warming embryo characteristics has not been evaluated in euploid embryos after preimplantation genetic testing for aneuploidy (PGT-A). In light of the limited evidence and conflicting results, we deem a need for a large study of single blastocyst transfer cycles in order to examine the value of post-warming embryo morphology in untested and euploid embryos.

In this cohort study of single vitrified blastocyst transfer cycles, we examined whether embryonal post-warming characteristics correlated with live birth. Furthermore, we aimed to determine whether certain characteristics imply very poor prognosis and should indicate discarding the embryo and warming another one, if available.

## Materials and methods

In this historic cohort study, we reviewed all single vitrified-warmed blastocyst transfer cycles performed in our center, between November 2016 and May 2017. Patients were then followed until delivery. We excluded cycles with donor eggs, surrogacy, or slow-frozen embryos. The study was approved by the local IRB (ID: 71/19-MIVF, July 23, 2019).

### Stimulation cycles and embryo culture

More than 90% of stimulation cycles were fixed antagonist cycles, in which gonadotropins were administered starting on cycle day 3, and GnRH antagonist was added on cycle day 8. The rest of the cycles included the administration of GnRH agonist for pituitary suppression, starting either in the mid-luteal phase of previous cycle (long agonist protocol) or day 2 of current cycle (flare-up protocol). hCG or GnRH agonist trigger was administered when at least one follicle reached ≥ 18 mm.

Embryos were cultured individually in 25 drops of medium GTL (Vitrolife) in 12-well dishes under Ovoil (Vitrolife) in MINCS under 5% O_2_ and 6% CO_2_. Time-lapse embryoscopy was not introduced into our laboratory until after the study period. PGT-A was performed in some of the cycles, as per patient request. Trophectoderm biopsy was performed, and cells were tested by NGS.

### Embryo vitrification and warming protocols

The Rapid-i closed vitrification system with RapidVit™ Blast solutions were used for blastocyst vitrification (Vitrolife). All procedures were carried out on a heated stage at 37 °C. Blastocysts were equilibrated in MOPS buffered media (Vitri 1 Blast), before 2 min a solution containing ethelene glycol and propandiol (Vitri 2 Blast), and then 35–45 s in Vitri 3 Blast. The blastocyst was then loaded into the Rapid-i device and vitrified.

All PGT-A blastocysts had assisted hatching performed via RI Saturn laser, with subsequent blastocoel collapse at the time of trophectoderm biopsy. PGT-A blastocysts that had re-expanded at time of vitrification post-trophectoderm biopsy exhibited blastocoel collapse again due to pipetting within the last vitrification solution. Non-PGT-A blastocysts did not have assisted hatching and also exhibited blastocoel collapse during pipetting at time of vitrification within the last vitrification solution.

For embryo warming, RapidWarm Blast™ (Vitrolife) solutions were used at 37 °C. The Rapid-i device was removed from liquid nitrogen and was directly placed into Warm 1 Blast. After 5 min, the blastocyst was moved to Warm 2 Blast solution for a further 5 min, followed by incubation in MOPS buffered media for 5 min. Blastocysts were then incubated in GTL under 5% O_2_ and 6% CO_2_ until transfer.

All embryos were warmed early in the morning, and the time of transfer differed according to doctor and patient preferences and availability. The exact times of warming and transfer were documented for each transfer.

Blastocyst survival was assessed according to post-warming morphologic appearance. If it was degenerated or less than < 50% cell survival, it was classified as “not surviving.” In this study, we included only cycles in which the blastocyst had survived the warming process and was subsequently transferred.

### Vitrified-warmed embryo transfer cycles

The majority of frozen embryo transfer (FET) cycles were natural cycles, in which a single blastocyst was transferred 6 days following the detection of LH surge. Artificial cycles included the administration of estradiol valerate (Progynova, Bayer Australia) 2 mg three times a day starting from cycle day 3, followed by the addition of progesterone supplementation when endometrial thickness reached ≥ 7 mm. Blastocyst transfer was performed after 5 full days of progesterone supplementation. Stimulated cycles included the administration of gonadotropins and embryo transfer 7 days following hCG trigger.

### Blastocyst grading

Before vitrification, blastocysts were graded according to the degree of expansion and trophectoderm quality, as previously described [6]. The degree of expansion was graded as 1, cavitating; 2, early blastocyst; 3, blastocyst; 4, expanding; 5, hatching; and 6, hatched. Trophectoderm grade was defined as good, fair, or poor.

**Fig. 1:**
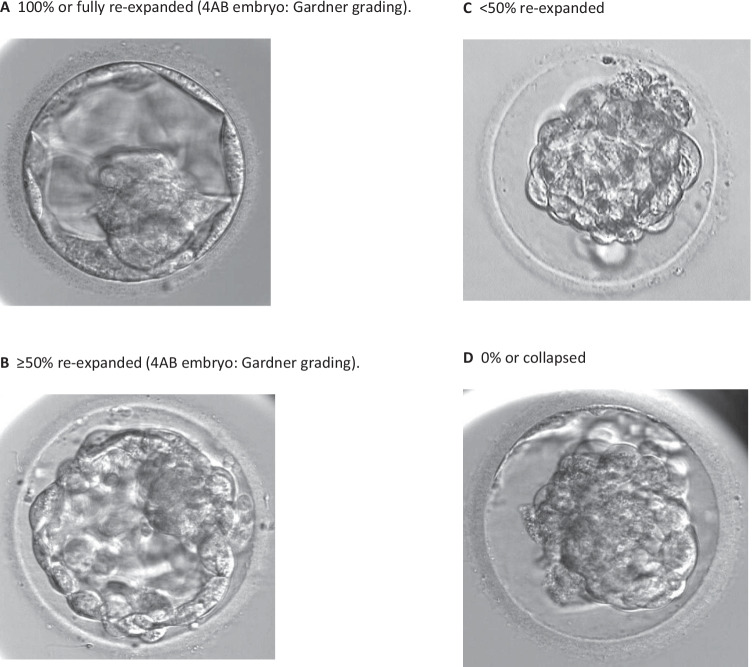
Grading of post-warming re-expansion in a pre-vitrification expanding (grade 4) blastocyst. **A** 100% or fully re-expanded (4AB embryo, Gardner grading). **B** ≥ 50% re-expanded (4AB embryo, Gardner grading). **C** < 50% re-expanded. **D** 0% or collapsed

Immediately before embryo transfer, a single observation was made by one of the participating embryologists to determine the degree of embryo re-expansion and cell survival. The degree of blastocoel re-expansion was graded in comparison to pre-vitrification degree of expansion as A, fully re-expanded; B, partially re-expanded ≥ 50%; C, partially re-expanded < 50%; and D, collapsed (Fig. 1). The degree of post-warming cell survival was graded on a scale of 50 to 100% and was later classified into 4 groups for statistical analysis: very low, 50–70%; low, 71–80%; moderate, 81–90%; and high, 91–100%. Embryologists estimated the degree of post-warming cell survival after warming and again immediately before transfer, by visually identifying intact cell membranes in comparison to degenerate apoptotic cells whereby cell membranes are porous and cellular contents have breached the compromised cell membranes.

The grading of embryos before and after vitrification and warming was done by a total of 32 experienced embryologists using an inverted microscope. All participating embryologists were trained within the same laboratory using the same laboratory protocols and guidelines. The first author additionally had one-on-one training with the participating embryologists, where the same embryos were assessed simultaneously to reduce interobserver variability while assessing post-warming re-expansion and cell survival.

### Data collection and clinical outcomes

For each patient, the following clinical data were retrieved from our database: female age on egg collection, infertility diagnoses (male, female, combined male and female, unexplained, or other), gravidity, fresh cycle protocol (long agonist, flare, or antagonist), number of eggs collected, number of frozen embryos, whether euploidy was tested with PGT-A, vitrification-warming cycle protocol (natural, artificial, or stimulated), maximal endometrial thickness during FET cycle, time interval between warming and embryo transfer, and cycle outcome.

The primary outcome was live birth rate (LBR) per transfer. Live birth was defined as a birth of a live infant ≥ 24 weeks of gestation. Secondary outcomes were positive hCG test, clinical pregnancy (defined as demonstration of fetal pole with cardiac activity on ultrasound scan), and miscarriage (defined as a non-viable pregnancy after demonstration of intrauterine pregnancy on ultrasound scan).

### Statistical analysis

Statistical analysis was performed using SPSS software V27 (IBM, USA). The Kolmogorov–Smirnov test was used for the assessment of normality. Continuous variables with and without normal distributions were presented as mean ± standard deviation (SD) or median (interquartile range), respectively. Continuous variables were compared using Student’s *t* test or Mann–Whitney *U* test, as appropriate. Nominal variables were compared using the chi-square test or Fisher exact test, as appropriate. Pair-wise comparisons were performed using the Bonferroni method. Multivariate logistic regression analyses were performed to examine the association between post-vitrification morphology and live birth (the dependent variable). Female age at egg collection (continuous), PGT-A (yes/no), pre-vitrification expansion (poor prognosis 1 or 2, moderate prognosis 3 or 6, good prognosis 4 or 5), and pre-vitrification trophectoderm grade (non-gradable, poor, fair, good) served as independent variables. Finally, we performed a sub-group analysis of embryos which were found euploid according to PGT-A. A post hoc power calculation showed that the study had 99.9% power to detect a difference in the live birth rate between collapsed and fully expanded embryos, with alpha of 0.05.

## Results

Overall, 612 single vitrified-warmed blastocyst transfer cycles were included. In 196 cycles, euploid embryos were transferred after PGT-A. The mean patient age on the day of egg collection was 35.7 ± 3.9 years (range 22.1–45.3). Immediately before transfer, 283 (46.2%) embryos were fully expanded, 156 (25.5%) embryos were partially expanded ≥ 50%, 103 (16.8%) embryos were partially expanded < 50%, and 70 (11.4%) embryos were collapsed. Median cell survival rate was 90% (range 50–100%). The live birth rate of all included FET cycle was 28.3%.

Table 1 presents patient characteristics according to whether or not they achieved a live birth in the vitrified-warmed embryo transfer cycle. Patients who achieved a live birth after FET had embryos with higher pre-vitrification expansion and trophectoderm grades.

**Table 1:** Patients’ characteristics according to whether or not they achieved a live birth in the vitrified-warmed embryo transfer cycle

	**No live birth (*n* = 439)**	**Live birth (*n* = 173)**	***p* value**
Female age on egg collection (years)	35.9 ± 4.0	35.4 ± 3.9	0.16
BMI (Kg/m^2^)	24.5 ± 5.1	24.2 ± 4.7	0.58
Nulligravida	315 (71.8)	120 (69.4)	0.55
Cause of infertility			0.24
Male factor	128 (29.2)	54 (31.2)	
Female factor	182 (41.5)	73 (42.2)	
Mixed male and female factors	59 (13.4)	17 (9.8)	
Unexplained	65 (14.8)	23 (13.3)	
Other	5 (1.1)	6 (3.5)	
Stimulation protocol			0.17
Antagonist	403 (91.8)	161 (93.1)	
Flare	17 (3.9)	2 (1.2)	
Long agonist	19 (4.3)	10 (5.8)	
Number of eggs collected	14 (8–20)	14 (10–20)	0.62
Number of 2 pn embryos	8 (5–12)	8 (6–13)	0.32
Number of frozen embryos	3 (2–6)	4 (2.5–6)	0.07
Day of vitrification			1.00
5	248 (56.5)	98 (56.6)	
6	191 (43.5)	75 (43.4)	
Expansion grade pre-vitrification			0.004
1	6 (1.4)	0	
2	50 (11.4)	7 (4.0)	
3	115 (26.2)	35 (20.2)	
4	222 (50.6)	109 (63.0)	
5	39 (8.9)	21 (12.1)	
6	7 (1.6)	1 (0.6)	
Trophectoderm grade pre-vitrification			< 0.001
Non-gradable	22 (5.0)	2 (1.2)	
Poor	72 (16.4)	10 (5.8)	
Fair	167 (38.0)	61 (35.3)	
Good	178 (40.5)	100 (57.8)	
PGT-A	116 (26.4)	80 (46.2)	< 0.001
FET cycle protocol			0.78
Natural	276 (62.9)	114 (65.9)	
Artificial	99 (22.6)	36 (20.8)	
Stimulated	64 (14.6)	23 (13.3)	
Endometrial thickness pre-FET (mm)	9 (8–9.5)	9 (8–9)	0.79
Time interval between warming and transferring the embryos (hours)	3.2 (2.6–4.0)	3.4 (2.7–4.2)	0.16

Data are presented as mean ± SD, median (interquartile range), or *n* (%)

*FET*, frozen embryo transfer

Table 2 presents FET cycle outcomes according to the degree of post-warming embryo re-expansion. The rates of positive hCG test, clinical pregnancy, and live birth significantly increased as the degree of re-expansion increased. Miscarriage rate did not differ between the groups.

**Table 2:** Outcomes of single vitrified-warmed blastocyst transfer cycles according to the degree of post-warming embryo re-expansion

	**Collapsed (*n* = 70)**	**Partial re-expansion < 50% (*n* = 103)**	**Partial re-expansion ≥ 50% (*n* = 156)**	**Full re-expansion (*n* = 283)**	**Unadjusted *p* value**
Positive hCG	14 (20.0) ^a^	32 (31.1) ^a, b^	61 (39.1) ^b^	164 (58.0)	< 0.001
Clinical pregnancy	8 (11.4) ^a^	18 (17.5) ^a, b^	43 (27.6) ^b^	127 (44.9)	< 0.001
Miscarriage	1 (1.4)	8 (7.8)	6 (3.8)	23 (8.1)	0.09
Live birth	8 (11.4) ^a^	15 (14.6) ^a^	40 (25.6) ^a^	110 (38.9)	< 0.001

Data are presented as *n* (%)

Values with the same superscript letter did not differ significantly in per-wise comparisons (*p* > 0.05)

**Fig. 2:**
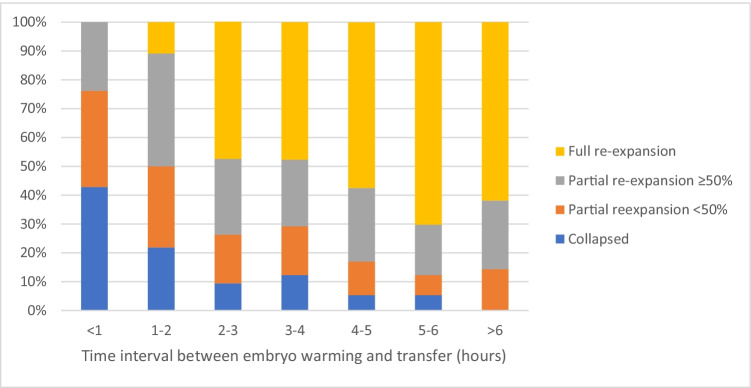
Degree of blastocyst re-expansion according to time interval between warming and transfer. *p* < 0.001 (unadjusted)

The median time interval between warming and transfer of an embryo was 3.3 h (range 0.2–7.7). The degree of re-expansion was higher as the time interval between warming and transfer was longer (divided into groups of 1 h, *p* < 0.001) (Fig. 2). However, warming-transfer interval was not correlated with live birth (*p* = 0.53). Among the 535 embryos that were transferred after 2 h or more, 49 (9.2%) remained collapsed, whereas 279 (51.6%) were fully re-expanded. The live birth rate was 8.2% for collapsed embryos and 39.1% for embryos that were fully expanded after 2 h or more (*p* < 0.001).

Table 3 presents FET cycle outcomes according to the degree of post-warming cell survival. The rates of positive hCG test, clinical pregnancy, and live birth significantly increased as the degree of post-warming cell survival increased. Miscarriage rate did not differ between embryos with different cell survival rate. When examining embryos with the worst post-warming characteristics, 15 collapsed embryos with only 50–70% cell survival rate were transferred, out of which one transfer (6.7%) resulted in a live birth at 38 weeks of gestation. PGT-A had not been performed on this embryo. In contrast, 177 embryos had the best post-warming characteristics, namely, full re-expansion and cell survival rate > 90%, out of which 71 (40.1%) transfers resulted in a live birth (Supplementary Table 1).

**Table 3:** Outcomes of single vitrified-warmed blastocyst transfer cycles according to the degree of post-warming cell survival

	**50–70% cell survival (*n* = 31)**	**71–80% cell survival (*n* = 60)**	**81–90% cell survival (*n* = 218)**	**91–100% cell survival (*n* = 303)**	**Unadjusted *p* value**
Positive hCG	5 (16.1) ^a^	20 (33.3) ^a,b^	93 (42.7) ^b^	153 (50.5) ^b^	< 0.001
Clinical pregnancy	3 (9.7) ^a^	12 (20.0) ^a^	66 (30.3) ^a,b^	115 (38.0) ^b^	0.001
Miscarriage	2 (6.5)	2 (3.3)	17 (7.8)	17 (5.6)	0.57
Live birth	2 (6.5) ^a^	12 (20.0) ^a,b^	54 (24.8) ^a,b^	105 (34.7) ^b^	0.001

Data are presented as *n* (%)

Values with the same superscript letter did not differ significantly in per-wise comparisons (*p* > 0.05)

On multivariate logistic regression analyses, post-warming partial embryo expansion ≥ 50% (adjusted OR 2.37, 95% CI 1.02–5.51) and full embryo re-expansion (adjusted OR 4.40, 95% CI 1.99–9.72) remained significantly associated with live birth, after controlling for patient age, pre-vitrification expansion grade, pre-vitrification trophectoderm grade, and PGT-A (Table 4). When testing for cell survival rate, only high cell survival rate (91–100%) remained significantly associated with live birth, after controlling for the same variables (adjusted OR 5.22, 95% CI 1.19–22.78, *p* = 0.02). The time interval between warming and transfer was not associated with live birth after controlling for the same variables (adjusted OR 1.07, 95% CI 0.97–1.18, *p* = 0.16).

**Table 4:** Multivariate logistic regression analysis — odd ratios for live birth according to post-warming re-expansion grade

	**Adjusted OR (95% CI) **^**a**^	***p* value**
Female age	0.96 (0.91–1.01)	0.11
Pre-vitrification expansion grade	1.43 (1.01–2.03)	0.03
Pre-vitrification trophectoderm grade	1.51 (1.14–2.00)	0.001
PGT-A confirmed euploidy	2.12 (1.43–3.15)	< 0.001
Post-warming re-expansion grade		
Collapsed	1 (reference)	
Partial re-expansion < 50%	1.22 (0.48–3.13)	0.66
Partial re-expansion ≥ 50%	2.37 (1.02–5.51)	0.04
Full re-expansion	4.40 (1.99–9.72)	< 0.001

^a^Odds ratios and 95% confidence intervals. ORs are adjusted for all the variables that are included in the table

**Fig. 3:**
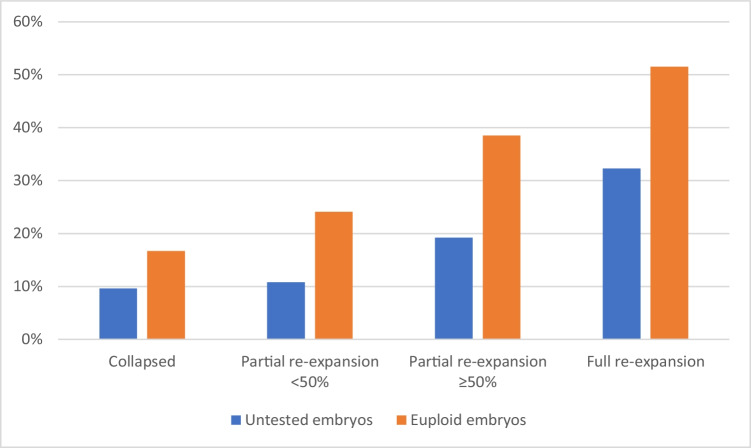
Live birth rate according to post-warming blastocyte re-expansion grade in untested and euploid embryos. *p* < 0.001 for untested embryos (unadjusted). *p* = 0.006 for euploid embryos (unadjusted)

Finally, we performed a sub-group analysis of the 196 euploid embryos according to PGT-A. Again, the LBR increased as post-warming re-expansion grade increased; from 16.7% in collapsed embryos to 51.5% in embryos with full re-expansion (*p* = 0.006). Figure 3 presents the LBR according to re-expansion grade in untested and euploid embryos. Moreover, in a sub-group analysis of only euploid embryos, the LBR was higher in embryos with high cell survival rate, compared to embryos with very low to moderate cell survival rate (48.5 vs. 32.6%, respectively, *p* = 0.02).

## Discussion

In this study, we found that post-warming blastocyst characteristics correlated with cycle outcome. Higher re-expansion grade and cell survival rate were associated with higher LBR, even when controlling for pre-vitrification characteristics and PGT-A. Similar results were found in a sub-group analysis of only euploid blastocysts. Nevertheless, blastocysts with poor post-warming morphology still demonstrated a considerable probability of live birth.

Blastocysts are typically collapsed immediately after warming. Therefore, a post-warming culture provides the opportunity to further assess embryo integrity. The ability to re-expand was previously found to indicate embryo potential. Already in 2009, Shu et al. [10] demonstrated in a study of 237 blastocyst transfer cycles that re-expansion and cell survival rates correlated with clinical pregnancy rate. In that study, non-expanded embryos were discarded, as they were considered embryos that had not survived. Re-expansion was later found to be associated with live birth as well [8, 11, 12]. Coello et al. reported the outcome of 429 frozen-warmed blastocysts transfer cycles in patients who received egg donation. Re-expansion was strongly correlated with implantation rate [11]. Our results are in agreement with these studies.

In contrast, a large retrospective study by Cimadomo et al. [9] reported that post-warming features were not associated with live birth when controlled for pre-vitrification parameters. However, in that study, blastocyst re-expansion was tested 1.5 h after warming, which might be too early for assessing the true rate of re-expansion. Furthermore, classification of embryos according to re-expansion rate was different than in the current study (full re-expansion 80–100%, partial re-expansion 20–80%, and no re-expansion 0–20%), and only 2.5% of all embryos were defined as “unexpanded.” Recently, Giunco et al. [14] have reported that post-warming blastocyst re-expansion did not affect clinical pregnancy rates. In this study, pictures of 486 embryos were taken after warming and again before transfer, and the longest portion of the embryo was measured for each picture. Expansion was defined as increase in measurement, not taking into account the degree of blastocoel formation. Moreover, laser-induced collapse was performed before vitrification. This might contribute to the differences between their findings and ours.

The process of blastocoel development and blastocyst expansion involves active transport of sodium ions by trophectoderm cells, followed by passive influx of water due to osmotic pressure [17]. Indeed, pre-vitrification grades of expansion and trophectoderm are correlated with live birth [6]. Furthermore, blastocysts with a higher developmental stage demonstrate higher level of glucose uptake [18]. Therefore, blastocyst re-expansion may reflect cell viability and embryo potential. A previous study [19] examined the ability of individual bovine blastocysts to survive freezing and thawing by measuring glucose and pyruvate uptake and lactate production. In the 5 h after thawing, those blastocysts that expanded their blastocoel had significantly greater glucose and pyruvate uptake and lactate production than those embryos that failed to develop. Finally, a previous study reported the morphokinetics of vitrified-warmed blastocysts by placing them in a time-lapse imaging system immediately after warming and until transfer [20]. It was found that earlier onset of re-expansion was predictive of pregnancy and live birth. These results further emphasize that the process of post-warming re-expansion is a marker of embryo integrity. Unfortunately, in the current study, embryos were graded immediately before transfer, and the time interval between warming and transfer was variable. Hence, the timing of re-expansion onset was not recorded.

The American Society for Reproductive Medicine (ASRM) defined “futile” treatment as a treatment with < 1% chance of achieving a live birth and “very poor prognosis” treatment as a treatment with 1–5% chance of achieving a live birth per cycle [21]. In the current study, we found that transferring embryos that remained collapsed after warming resulted in a LBR of 11.4%, and transferring embryos with only 50–70% cell survival rate resulted in a LBR of 6.5%. When analyzing embryos with the worst post-warming morphology (collapsed with 50–70% cell survival), a LBR of 6.7% was noted. Therefore, we concluded that embryos should not be discarded based only on post-warming morphology.

We suggest that post-warming blastocyst characteristics may serve four purposes. First, in centers where the common practice is to transfer more than one embryo in poor-prognosis patients, post-warming morphology can help making the decision on when to warm and transfer another embryo. A small risk of multiple pregnancy, however, cannot be eliminated. Second, post-warming parameters can help counsel patients regarding their embryo quality and probability of live birth. Third, post-warming re-expansion and cell survival may serve as laboratory performance indicators, with the aim of improving vitrification and warming procedure as well as culture conditions. Fourth, post-warming parameters may serve as a research tool, by which one can estimate the safety and efficacy of new laboratory procedures, techniques, and devices.

The strengths of the current study are the large number of cycles and the policy of single blastocyst transfer. Furthermore, we examined the significance of post-warming parameters in a sub-group of euploid PGT-A tested embryos. Nevertheless, several limitations should be acknowledged; first, during the study period, we did not grade the embryo inner cell mass. Hence, full Gardner score could not be presented. Second, the time interval between warming and transferring an embryo was variable. However, it was comparable between cycles that had and had not resulted in a live birth. Moreover, the time interval between embryo warming and transfer was found not to affect reproductive outcome [22]. Third, there was a significant heterogeneity in the characteristics of the studied cycles, including the use of PGT-A in some, but not all, cycles. In order to adjust for some of the possible confounders, we used a multivariate analysis. Finally, the study was performed in 2016–2017. Since then, our laboratory and clinical practice have been greatly improved. However, practice was consistent during the study period, and the results should reflect the real impact of post-warming morphology.

In conclusion, post-warming re-expansion and high cell survival rate are associated with a higher LBR. However, blastocysts with poor post-warming morphology still demonstrate a considerable probability of live birth. Therefore, post-warming characteristics can help improving patient counseling and laboratory efficiency, but poor morphology cannot indicate discarding an embryo.

## Supplementary Information

Below is the link to the electronic supplementary material.

Supplementary file1 (DOCX 18 kb)
