# Supplementary material for: Post-warming embryo morphology is associated with live birth: a cohort study of single vitrified-warmed blastocyst transfer cycles
Source: J Assist Reprod Genet. 2022 Jan 18;39(2):417–25. doi: 10.1007/s10815-021-02390-z (PMC8956752; doi:10.1007/s10815-021-02390-z)
Supplement: Supplementary file 1 — Supplementary file1 (DOCX 18 kb) [file 10815_2021_2390_MOESM1_ESM.docx]

Supplementary table 1. Live birth rate according to post-warming blastocyst re-expansion and cell survival.

|  | **Degree of re-expansion** | | | | |
| --- | --- | --- | --- | --- | --- |
| **Cell survival rate** |  | Collapsed | Partial reexpansion <50% | Partial reexpansion ≥50% | Full reexpansion |
|  | 50-70% | 6.7% | 20.0% | 0.0% | 0.0% |
|  | 71-80% | 23.1% | 4.8% | 8.3% | 50.7% |
|  | 81-90% | 7.4% | 14.3% | 23.7% | 35.6% |
|  | 91-100% | 13.3% | 20.0% | 32.9% | 40.1% |
